# Health professionals’ perceptions of prehabilitation before haematopoietic cell transplantation to optimise candidacy in older adults

**DOI:** 10.1007/s00520-024-08659-0

**Published:** 2024-06-26

**Authors:** E. Guinan, C. Heuston, G. Sheill, M. Ní Chonghaile, N. Orfali

**Affiliations:** 1 Discipline of Physiotherapy, Trinity College Dublin, Dublin, Ireland; 2 Trinity St James’s Cancer Institute, Dublin, Ireland; 3 Department of Physiology, Trinity College Dublin, Dublin, Ireland; 4 Department of Physiotherapy, St James’s Hospital, Dublin, Ireland; 5 National Adult Stem Cell Transplant Unit, St James’s Hospital, Dublin, Ireland

**Keywords:** Haematopoietic stem cell transplant, Frailty, Gerontology, Prehabilitation, Haematologic malignancy

## Abstract

**Purpose:**

Haematologic malignancies for the most part are diseases of the elderly. Haematopoietic stem cell transplantation (HSCT) remains the only potentially curative strategy for many patients but carries substantial morbidity and mortality risks, particularly in frail or co-morbid patients. Pre-transplant optimisation of key targets through prehabilitation may have significant clinical impact.

**Methods:**

We utilised qualitative methodology (semi-structured interviews) to gain insights and understanding of the perceptions of medical, nursing and allied health professionals towards prehabilitation before haematopoietic cell transplantation to optimise candidacy in older adults. Thematic analysis was performed using a qualitative descriptive approach completed in duplicate by two researchers.

**Results:**

Between August and October 2023, eleven health professionals participated from four large cancer centres across the island of Ireland (*n* = 3 consultant haematologists, *n* = 7 specialist haematology nurses and *n* = 1 senior haematology physiotherapist). Four major themes were identified. The themes *comprehensive biopsychosocial care* and *increasing demand for transplant in older patients* highlight the unique challenges impacting older adults who receive HSCT. The *multimodality pathways of care* theme highlights the heterogeneity of treatment pathways across different clinical sites and disease types. This has implications for the *prehabilitation: logistics and benefits* theme, which indicated strong support for prehabilitation but emphasised that implementation must consider national reach and context.

**Conclusions:**

There is broad national multidisciplinary interest in the development of prehabilitation programmes for patients being considered for transplant. Our results will inform the development of services in this area in consideration of national reach, malignancy-specific pathways and the unique factors associated with older age.

## Introduction

Therapeutic advances for haematological cancers have improved survival rates; however, treatment is often associated with significant toxicity and prolonged hospitalisation, which may negatively affect patients’ quality of life and physical function [1]. In particular, older adults with haematological cancer have a poorer prognosis than younger adults and suffer higher treatment-related morbidity and mortality [2]. As haematologic malignancies have the highest incidence in people aged ≥ 65 years, optimising treatment options for this cohort is essential.

Haematopoietic cell transplantation (HSCT) offers a potentially curative option for patients with high-risk or relapsed/refractory disease [1]. Autologous HSCT involves the administration of high-dose chemotherapy which aims to eradicate disease and ablate the bone marrow, followed by a stem cell rescue using a patient’s own cells pre-harvested and cryopreserved. In allogeneic HSCT, donor stem cells are used to replace diseased marrow with healthy haematopoiesis, while also generating a new donor-derived immune system that can mount a “graft versus tumour” effect. Despite advances, HSCT remains a high-toxicity treatment with a substantial risk of severe complications including death [3]. HSCT involves protective isolation requirements pre- and post-transplant due to immunosuppression, often resulting in prolonged periods of reduced physical activity. Combined with toxicity mediated by chemo-/radio-/immunosuppressive therapy, patients are at a higher risk of developing long-standing physical and psychosocial problems such as pain, muscle atrophy and cancer-related fatigue [4]. In addition, graft versus host disease, a systemic disorder that occurs when the graft’s (transplanted/donated) immune cells recognise the host (the recipient) as foreign and attack healthy recipient tissue, is commonly observed in recipients after allogeneic HSCT and is a substantial risk factor in terms of loss of physical functioning, fatigue and depression mostly due to steroid-induced myopathy [5, 6].

Older age at diagnosis coincides with the accumulation of geriatric conditions and co-morbidity, presenting unique challenges for patients and clinicians alike [7, 8]. Treatment decisions are often more difficult in the elderly, where frailty can pose a substantial barrier to curative strategies [9]. With the increasing availability of novel targeted drugs and immunotherapeutic agents in the haematology arena, older patients with haematologic cancer are attaining higher rates of disease remissions. These regimens are associated with less toxicity than standard chemotherapy, allowing a greater number of older patients to be considered as transplant candidates [8]. In parallel, increased international experience with reduced-intensity conditioning platforms has reduced the risks associated with transplantation. In this era, advanced chronologic age no longer represents an absolute contra-indication to transplantation [10, 11]. Instead, multidisciplinary input into selecting and optimising candidacy is preferred and an understanding of the unique physical challenges facing the elderly cohort is required.

Prehabilitation is a process on the continuum of care that occurs between the time of cancer diagnosis and the beginning of treatment, includes physical and psychological assessments that establish a baseline functional level, identifies impairments and provides targeted interventions that improve a patient’s health to reduce the incidence and the severity of current and future impairments [12]. Prehabilitation in solid tumours is widely used for pre-operative optimisation of physical, nutritional and psychological indices [13, 14]. Pre-transplant optimisation of key targets through prehabilitation may have a significant clinical impact; however, the role of prehabilitation in haematological malignancies is under-investigated, and implementation challenges or success factors are poorly understood. This study aimed to gain insights and understanding of the perceptions of medical, nursing and allied health professionals towards prehabilitation before HSCT to optimise candidacy in older adults.

## Methods

### Study design and sampling

A qualitative methodology using semi-structured interviews was utilised. Participants were recruited using purposive sampling methods targeting medical, nursing and allied health professionals working in cancer centres on the island of Ireland. Ethical approval was granted by Trinity College Dublin School of Medicine Research Ethics Committee in June 2022 (reference number 2562). This work was completed in accordance with the Declaration of Helsinki. Those interested in participating contacted the study team directly and provided written informed consent prior to the interviews. Findings are reported as per the COnsolidated criteria for REporting Qualitative research (COREQ) checklist [15].

### National context for haematopoietic stem cell transplant in Ireland

The research team are based at St James’s Hospital in Dublin—Ireland’s largest haematology cancer centre which houses the designated National Adult Allogeneic Transplant Programme and an Autologous Stem Cell Transplant Program. The service is Joint Accreditation Committee ISCT-Europe & EBMT (JACIE)–accredited and is the third largest SCT unit in the UK and Ireland.

### Data collection and interview topics

Semi-structured interviews were conducted face-to-face, by telephone or through videoconferencing as convenient for the participant between August and October 2023. Interviews were conducted by a female researcher, lecturer and physiotherapist (EG), who has over 15 years of experience in cancer rehabilitation and survivorship, including extensive work in surgical prehabilitation. Most interviews were also attended by student CH, who noted observations.

Participants were informed of the goals of the research in advance of participation by standardised email and participant information leaflets distributed by MNíC, Transplant Co-ordinator at the National SCT, with over 30 years of haematology nursing experience.

Details pertaining to professional grade and experience with haematopoietic cell transplantation were documented. Data were collected through semi-structured interviews using a flexible interview guide which examined the following broad topics:

1. What are your perceptions or views of the impact of older/chronological age on transplant candidacy?
2. What do you believe are the important factors that should be assessed in order to determine transplant candidacy?
3. What do you believe is the role of prehabilitation in optimising older patients’ physical condition in advance of transplant?
4. Do you have any recommendations for research questions in this area?

The interview guide was developed by EG with feedback from a Consultant Haematologist (NO) and Clinical Specialist Physiotherapist in Cancer Rehabilitation (GS). Interviews were audio-recorded to an institutional Zoom Video Communications account, immediately transcribed verbatim and pseudonymised.

### Data analysis

Pseudonymised transcripts were imported into NVivo 20 qualitative data analysis management software (QSR International, Melbourne, Australia). Transcripts were analysed independently by two reviewers (EG and MNíC). Analysis was completed using an inductive thematic approach [16]. This approach allows for a data-driven examination of the interview data, complementing the exploratory nature of this work, and providing scope for unique insights and experiences to be acknowledged. In the first step, EG and MNíC engaged in a period of familiarisation involving reading and re-reading the transcripts, and meeting to discuss initial thoughts. Following that, codes were independently generated, and illustrative quotes were identified which were related to the research aim. EG and MNíC met to discuss coding analysis, with the aim of developing and prioritising codes that best reflected the analysis. Codes were grouped into themes and final themes were agreed through consensus. EG and MNíC reflected throughout the process on how our experiences shaped the interpretation of the dataset. Using multiple coders with different background experiences (one physiotherapy academic experienced in surgical prehabilitation and one nurse specialist with over 30 years of experience in HSCT) encouraged exploration of the codes and themes through a multidisciplinary lens that was cognisant of national and international HSCT service delivery challenges and relevant literature.

## Results

**Fig. 1:**
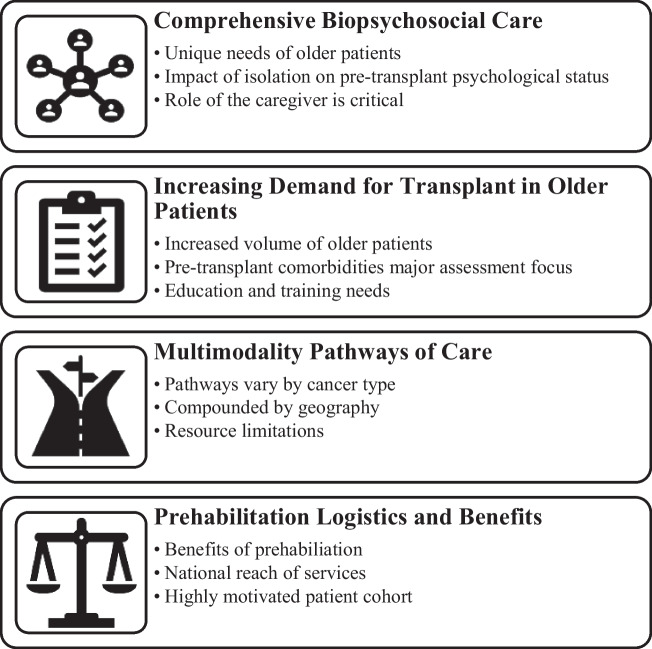
Themes and sub-themes identified from semi-structured interviews

Twenty-one letters of invitation were distributed. Eleven health professionals from four cancer centres across the island of Ireland (St James’s Hospital, Tallaght University Hospital, University Hospital Cork, Belfast City Hospital) participated. Participants included consultant haematologists (*n* = 3), specialists haematology nurses including advanced nurse practitioners (*n* = 2), transplant coordinators (*n* = 3), clinical nurse specialists (*n* = 1), a transplant ward clinical nurse manager (*n* = 1) and a senior haematology physiotherapist (*n* = 1). Participants worked across pre-transplant assessment, in-patient care, late effects clinics and rehabilitation services. Participants estimated that they reviewed patients aged ≥ 60 for transplant, at most, up to 10 times per week, across the pre- and post-transplant settings. Two interviews were conducted in-person, one by telephone and seven using Zoom Video Communications. Interviews lasted 17–38 min (average 27 min 30 s). Four major themes, each with three sub-themes were identified (Fig. 1).

### Comprehensive biopsychosocial care

#### Unique needs of older patients

Many participants spoke about the value of tailored care pathways to meet the needs of older patients. Participants gave the example of the age-appropriate infrastructure and staffing within the transplant unit for adolescent and young adult (AYA) cancer patients and how effective these resources were for improving the quality of care that they could offer patients.

“…recently we have opened up a four-bedded AYA unit here … we have an AYA team as well as our haematology team and it’s phenomenal, but it needs to be across the board…. this level of care should be across all of our age groups and particularly our older age group who really require, probably geriatrician input…” (HRBSS2301).

“.. older people should have a clinical nurse specialist when the patient cohort increases. So there will be a clinical nurse specialist who will be dealing with their own problem because even in the discharge advice, sexuality and fertility is a topic that we discuss, it’s different for the older people….” (HRBSS2309).

#### Impact of isolation on pre-transplant psychological status

The psychological impact of prolonged isolation on patients undergoing HSCT was widely acknowledged. Participants spoke about the need for patients to be psychologically ready for the procedure but also about the toll that isolation can take on mood, activity participation and ability to cope while in hospital. When asked about future research priorities in this area, psychological preparation was a dominant feature.

“I think psychologically there’s a huge gap in preparing people for transplant. And certainly, here there’s very little support we can offer them. And I think it’s an area that people suffer from hugely post-transplant as a result.” (HRBSS2304).

“Assessing (psychological well-being) pre-transplant, some sort of initiative whereby they are supported psychologically throughout the transplant and post-transplant,” (HRBSS2307).

#### The role of caregivers is critical

The critical role of caregivers, particularly family members, in supporting patients prepare for and recover from HSCT, was broadly acknowledged. Advancing age in partner-caregivers was noted as an issue. High-quality carer support was felt to benefit the patient’s physical and emotional recovery.

“I see someone with a significant other or a spouse that’s there, I immediately take a deep breath”. (HRBSS2305).

“…the caregivers are older, that’s something…. Caregivers need to be with these patients probably for a year after transplant…. And they may or may not have caregivers at this stage of their lives, depending on, if it’s a partner or if it’s children, they may not be as available. (HRBSS2301).

### Increasing demand for transplant in older patients

#### Increased volume of older patients

Interviewees were unanimous that the age profile of patients receiving HSCT in recent years had increased and that chronological age was no longer a barrier to receiving HSCT. This was viewed positively however the issues of medical complexity with increasing age were acknowledged, particularly in relation to co-morbidities, which was the focus of pre-transplant assessment.

“We’re coming across far more patients with diabetes, with pre-existing heart conditions. And, early dementia, joint problems, you know, all of the things that would come with age”. (HRBSS2301).

“the main things are their comorbidities and, we work them up for transplant. So we do check their heart, lungs, kidneys, do ultrasound appointments, check their liver and things, make sure that there’s nothing there. (HRBSS2304).

#### Pre-transplant co-morbidities are the major assessment focus

Formal pre-transplant assessment of physical performance was rarely completed. Many consultant haematologists and transplant co-ordinators relied on informal assessments in this domain, such as observing patient mobility and independence with general activities.

“we still have this eyeball test that when you, call somebody in from the waiting room, you make a judgment on whether this is somebody who you could see getting through a treatment and their degree of fitness” (HRBSS2306).

#### Education and training needs

Finally, some staff discussed that the changing patient profile generated different learning needs for staff. Nurses spoke about the need for education in the care of older patients and the value of collaboration with geriatric services to leverage their clinical expertise.

“I never worked in geriatrics, but I’m learning a lot. It’s probably a piece of our education that we need to think about…. this new layer, we have an older patient with different requirements, different needs”. (HRBSS2301).

### Multimodality pathways of care

#### Pathways vary by diagnosis

Participants described how care pathways in haematology vary by cancer type and are further influenced by individual patient assessment, procedures from different referring hospitals, finding a suitable donor and capacity at the national allogenic HSCT centre. This may pose a challenge to introducing a standardised prehabilitation pathway as waiting times for HSCT may vary considerably.

“there’s a big lead in to transplant and then suddenly it’s a very short lead in and that’s not consistent…” (HRBSS2308).

“It takes variable lengths of time, and it could be something from three, four months until over a year.” (HRBSS2303).

#### Pathways are compounded by geography

The national reach of the services at St James’s Hospital is a key consideration in how services should be designed, particularly for older adults dependent on family members to travel considerable distances to attend in-person appointments. Coordinating all hospital appointments on the same day was proposed but this needs to be pragmatic. Prehabilitation service can learn from the challenges experienced in post-transplant pathways.

“…I think the 1:1 initial assessment would ideally be done face-to-face and could be done in clinic when they’re coming in for another appointment and then do a virtual online prehab class” (HRBSS2302).

“they can’t drive for the first few months at least after the transplant. So their independence is being affected, especially in the older people…. They have to depend mostly on the children and the children will be grown up as well at that stage, they have their own family and children and they have to find time to accompany the people to the post-transplant clinic” (HRBSS2309).

#### Resource limitations

The resources needed to deliver prehabilitation services could impact the breadth and potential flexibility that a prehabilitation programme could offer. Resources included time, space and personnel. In a resource-limited health service, prioritising prehabilitation for older adults scheduled for HSCT over younger patients scheduled for HSCT was considered necessary.

“I would focus on the older patients. But if there was time and time wasn’t a question and it was, let’s say a full-time post and or it had there was a staff grade who could assist and there could be more in the class or whatever it is, or a second class” (HRBSS2302).

### Prehabilitation logistics and benefits

#### Benefits of prehabilitation

The concept of prehabilitation prior to HSCT was enthusiastically welcomed by all interviewees. Many spoke about prehabilitation as a *no brainer*. While some did query if metrics, such as improved fitness, would have a major impact on HSCT outcomes, many regarded it as a straightforward concept that preparing patients in advance of such a major treatment could only have positive impacts.

“it’d be lovely to know that in somebody who’s doing well that there is something proactively done to try and improve their fitness and chances after”. (HRBSS2306).

“I think it can only, it can only help and… it won’t guarantee that the outcome will necessarily be any better just, because the virus reactivation, you know the graft versus host disease, is not controlled by fitness” (HRBSS2310).

#### National reach of services

Given the national context of the transplant services at St James’s Hospital, a prehabilitation programme would have to provide a service with a national geographic reach. Suggested models of care included virtually delivered or hybrid programmes.

“I suppose they’re so tied up in the referring hospitals, it would be very difficult even if they were from Dublin to get to a prehabilitation program in-person” (HRBSS2311).

“We’ve quite a large geographical spread so any kind of prehab to reach that volume of patients would need to be, I suppose at a minimum hybrid, but certainly have some sort of virtual option that people are able to join into”. (HRBSS2304).

Notwithstanding the benefits of virtually delivered or hybrid programmes, drawing on experiences from other rehabilitation programmes, digital literacy and access can pose a barrier to telerehabilitation, and mitigating solutions are needed.

(Question posed: What barriers may people experience?) “One if they have access to tablets. Which we have come across. But you know that that can be addressed. I’ve done pulmonary rehab before where we’ve had a stack of tablets where we’ve lent to patients, where we’ve gotten family members to help them log in”. (HRBSS2303).

#### Highly motivated patient cohort

Participants spoke confidently about the motivation and engagement of this patient cohort with all aspects of their care. Older patients scheduled for HSCT were described as highly motivated. It was widely agreed that an intervention such as prehabilitation would be very well received by this group of patients.

**“**I think their engagement is always really, really good and they’re always very keen to rehab.” (HRBSS2302).

“They’re fighting for their life to try and recover from this. So they’re very much engaged in it”. (HRBSS2305).

## Discussion

Geriatric haematology is a growing field and chronologic age alone is no longer a barrier to potentially curative therapies including HSCT. Our results indicate that there is broad national multidisciplinary interest in the development of prehabilitation programmes for patients being considered for transplant, notwithstanding numerous potential disease-specific and service delivery challenges.

Support for prehabilitation amongst those interviewed was high and this is reflected by others [17]. This is particularly encouraging as our study included health professionals from across a range of disciplines who work along all time points of the treatment trajectory, a key factor for implementation success [18]. In recognition of the increasing volume of HSCTs in ageing populations with complex health needs [19], and the potential for cancer treatment in the months preceding HSCT to accelerate age-related processes [20, 21], there is a critical need to inform supportive care strategies that work in parallel with standard therapies to optimise patient condition and outcomes. Prehabilitation has significant potential in this regard. In solid tumours, growing evidence from randomised clinical trials shows that structured prehabilitation can optimise pre-operative risk factors such as cardiopulmonary fitness or nutritional status, thus blunting the impact of surgery on patient condition, enhancing postoperative outcomes and improving health-related quality of life [22–27]. In HSCT, prehabilitation research is limited to small studies; however, emerging data are positive in respect to feasibility outcomes in addition to physical and psychological indices [28–31]. In older adults scheduled for HSCT, standardised assessments of physical frailty, geriatric assessment and patient-reported functionality assessments can be predictive of transplant outcomes including overall survival [32–37], non-relapse mortality [37, 38] and hospital length of stay [38–40], and therefore indicate amenable targets for pre-transplant optimisation through prehabilitation. Results from our interviews also strongly support a role for psychological preparation for transplant in older adults and identify pre-transplant psychological assessment and optimisation as a research priority. Further work that harnesses the support of the multidisciplinary team to examine the potential for prehabilitation in this setting is warranted. The Prehab4Cancer model in the UK provides an excellent example of how this is achieved through a stepped-care framework for intervention ranging from universal to specialists, which was developed to be collaborative, system-wide and scalable [41].

Implementation of prehabilitation programmes in surgical oncology is complicated by short timelines for implementation within surgical pathways, and significant concurrent patient burden associated with multiple hospital assessment, high-symptom burden and pre-operative anxiety [18, 42]. This burden is complex, but worthwhile, and overcoming individual barriers to prehabilitation is recommended [43]. Prehabilitation in HSCT brings unique implementation challenges [17]. Our results highlight significant pathway variation across haematological malignancies, and factors such as identifying stem cell donors could lead to protracted and unpredictable waiting times for HSCT. The challenge therefore lies in delivering prehabilitation programmes that are sufficient to optimise physical and psychological condition in advance of transplant but are delivered within a timeframe that supports optimal patient engagement. Notwithstanding these challenges, there was overwhelming confidence that older patients scheduled for HSCT are highly motivated and an anticipation of a high level of engagement with prehabilitation.

Older age is associated with an accumulation of geriatric conditions, and as reflected in our interviews, an increasingly complex patient profile in relation to assessment for transplant candidacy and post-transplant management [2]. Consequently, co-morbidity assessment comprised the major focus for pre-transplant evaluation. While emerging evidence suggests a role for geriatric assessment and validated measures of physical frailty as predictive of transplant outcomes in older adults [32–40], these outcomes were rarely, if ever, formally incorporated into routine practice at these Irish sites. Routine integration of these outcomes into clinical care pathways would aid the development of individualised prehabilitation programmes that target priority impairments.

In Ireland, all allogenic transplants are performed at the National Adult Allogeneic Transplant Programme at St James’s Hospital, thus providing service to the entire country (84,421 km^2^). This is similar to other jurisdictions where allogenic HSCTs are performed in Centres of Excellence serving large geographical areas [44]. The implications for prehabilitation services were clearly articulated by interviewees who advocated national focus and hybrid care models to reach all patients. The COVID-19 pandemic had changed telehealth in cancer care from a position of *novelty to normality* [45]. Notwithstanding the benefits of telehealth in terms of reach, convenience and burden, issues regarding accessibility and the value of in-person care appointments are noted [45]. In the current study, interviewees highlighted issues of digital literacy with older patients and dependency on family members for transport to in-person appointments are logistical barriers to implementation. Given these challenges, public and patient involvement [46] and collaborative working with multidisciplinary stakeholders should have a significant role in the design of prehabilitation programmes to optimise participant enrolment and retention [47].

There are some limitations to this work that warrant discussion. The sample size is small; however, the recruitment rate exceeded 50% from this small cohort of national experts and is drawn from four large haematology centres across the island of Ireland. Furthermore, the sample includes highly experienced haematology professionals, very familiar with the national landscape, from across a range of disciplines. This interdisciplinarity was reflected in the data analysis which allowed the data to be interpretated from multiple perspectives. While the context is specific to Ireland, learnings are applicable to other Centres of Excellence service large geographical areas. The research team is based at St James’s Hospital, Dublin, where surgical prehabilitation pathways are well established (> 800 referrals annually); however, prehabilitation and indeed rehabilitation pathways for haematological cancers are in their infancy. Consequently, the interview guide explored the issues from a broad perspective and in consideration of our national context. Future work would benefit from exploring specific contextual issues impacting implementation through an implementation science lens, utilising a framework such as the Consolidated Framework for Implementation Research [48].

In conclusion, health professionals recognise that prehabilitation has considerable potential to optimise outcomes for older adults scheduled for HSCT. Unique challenges related to the older adult, malignancy-specific pathways and service provision through national centres of excellence will influence the structure and delivery of prehabilitation programmes in this cohort. Ongoing and future efforts in this field should leverage the considerable support from multidisciplinary colleagues for prehabilitation and consider patient-centric models of care.

## Data Availability

Data are available on request to the corresponding author.
